# Morphological Measurement of Supracondylar Femur Based on Digital Technology in Chinese Han Population

**DOI:** 10.1111/os.12443

**Published:** 2019-04-16

**Authors:** Lei Wang, Dong‐mu Tian, Xin Liu, Jian‐feng Zhang, Li‐ming Zhao, Xin He, Yong‐cheng Hu

**Affiliations:** ^1^ Graduate School of Tianjin Medical University Tianjin China; ^2^ Department of Trauma Second Hospital of Tangshan City Tangshan China; ^3^ Beijing Weigao Yahua Artificial Joint Development Co. Ltd Beijing China; ^4^ Department of Rehabilitation Tangshan City Worker's Hospital Tangshan China; ^5^ First Department of Orthopaedics Xiqing Hospital Tianjin China; ^6^ Department of Orthopaedic Oncology Tianjin Hospital Tianjin China

**Keywords:** Anatomy, Femur, Knee, Morphology, Prosthesis

## Abstract

**Objective:**

Relatively few studies have reported on the morphology of the supracondylar femur, which is a fundamental factor affecting prosthetic reconstruction. The objectives of the present study were to measure the morphological parameters of the supracondylar femur, to classify the supracondylar femur, and to provide theoretical guidance for the development of distal femoral prostheses.

**Methods:**

The study consisted of 82 patients of Han Chinese nationality in North China. There were 57 men and 25 women included in the study, with an average age of 50.9 years (range, 18–87 years). Effective CT data should include a range of more than 10 cm for the distal femur. CT data for the right distal supracondylar femurs was obtained from DICOM files. Results for the cancellous bone and marrow cavity were retained, and information for the cortical bone was erased to obtain information of the lumen. Measurements of the intracortical cavity have not been reported previously. Lumen models were reconstructed with Mimics 17.0 software. The surfaces of the lumen models were smoothed with Geomagic studio 12.0 software. Using the Solidworks 2014 software, we established a 3‐D coordinate system, where variables of the lumen were examined. Correlations between the various measurements were calculated.

**Results:**

The supracondylar region of the femur was divided into five levels, and the length, breadth, height, and angle values were measured at each level. There were strong correlations between the length indexes (transverse diameter [EF], medial anteroposterior diameter [AC], middle anteroposterior diameter [GH], and lateral anteroposterior diameter [BD]) and the volume index (V). There were also strong correlations among the length indicators (EF, AC, GH, and BD) in each layer. Angle γ was correlated with the lateral anteroposterior diameter (BD) at L2–L6 layers (*r* = −0.383, −0.385, −0.296, −0.258, −0.24; all *P* < 0.05) and with the height index (h) at L4–L6 layers (*r* = −0.244, −0.385, −0.506; all *P* < 0.05). The most representative parameters were the medial anteroposterior diameter (AC_2_R_2_ = 0.865; AC_6_R_2_ = 0.932), the coronal width ratio, and the sagittal width ratio with volume. The analysis found that the lumen shape of flower–top hat accounted for 81% at most.

**Conclusions:**

The supracondylar femur has an asymmetrical structural area. The coronal plane is dominated by a flowerpot‐like morphology, and the sagittal plane is narrowest in the lateral 1/3 and resembles a top‐hat‐like morphology. Our results provide theoretical guidance for developing distal femoral prostheses and for their clinical application.

## Introduction

In a retrospective study, Unwin *et al.* (1996) found that the most common bones replaced with prostheses were the distal femur (39.0%), the proximal femur (21.5%), the proximal tibia (17.0%), and the proximal humerus (10.3%)^1^. The incidence of prosthetic failure is significantly higher in the distal femur^2^.

Many studies have pointed out that more than 40% of bone defects are due to a prosthetic failure in the distal femur^1^, ^3^. In a retrospective study of 1001 cemented prostheses used as replacements after surgery for bone tumors, Unwin *et al.* identified aseptic loosening as the principal mode of failure of massive distal femoral replacements. The probability of a patient surviving aseptic loosening of the distal femur was 67.4% for 10 years^1^. In addition to the impact of bone defects, the uncemented prosthesis may utilize osteointegration to minimize aseptic loosening, but short‐stem fractures and stem‐ratio‐matched intramedullary morphology have become the root causes of failure^4^, ^5^. Moreover, in the long term, there is little or not enough cancellous bone in the distal femur to provide the necessary compression for osteointegration. Therefore, in the distal femur, the key factors to reduce prosthetic failure are to reduce bone defects and improve the morphological match of the prosthesis with the bone. With the improvement of technology, the supracondylar femur becomes an important place for bone reserves after the removal of a knee with a tumor.

At present, aware of the hazards of bone defects, researchers have sought to reduce the prosthetic failure caused by wide resection through technical improvements^6^, ^7^, ^8^, ^9^, ^10^, ^11^. However, morphological studies of the femoral supracondylar intramedullary lumen are rare. Previous morphological studies have focused on extracortical or medullary cavities^12^, ^13^, ^14^, ^15^, ^16^, ^17^, ^18^, ^19^, but the actual interface of distal femur intramedullary fixation lies in the inner lumen of cortex. We consider that the difference between the inner lumen of cortex and the traditional medullary cavities lies in the cancellous bone, which has a great influence on the bone prosthesis interface. Because of reports of both initial stability and biological growth effects, the gap between the prosthesis and the bone interface needs to be very small^20^, ^21^. Morphological mismatch will increase the failure of the prosthesis in the distal femur. However, Chinese and Asian authors have reported significant differences in bone parameters between East Asian and Caucasian populations. At present, all the prostheses used in China lack data for application in Chinese people. These differences hinder the advantages of prostheses and can result in additional damage^22^, ^23^, ^24^, ^25^, ^26^, ^27^, ^28^.

The primary purpose of this study was to measure the morphological parameters of the femoral supracondylar intramedullary lumen, and to differentiate the “cortical lumen” from the medullary cavity of the femur shaft. Secondary aims were to classify the supracondylar femur and to provide theoretical guidance for the development of distal femoral prostheses and their clinical applications.

## Patients and Methods

The Ethics Committee of Tianjin Hospital approved the study, and written informed consent was obtained from all enrolled.

The study population consisted of 82 patients of Han Chinese nationality in North China, including 57 men and 25 women with an average age of 50.9 years (range, 18–87 years). All CT data for healthy right knees were obtained during the contralateral limb trauma or examination. Effective CT data should include a range of more than 10 cm for the distal femur.

### *Inclusion Criteria*

The inclusion criteria included: patients who received bilateral knee CT examination due to left limb trauma or physical examination; physical health and normal development, no tumor history; no history of trauma fracture of right femur and right knee; epiphyseal plate closure; no severe osteoporosis; no serious bone hyperplasia; the CT scan included more than 10 cm in length of the distal right femur.

### *Exclusion Criteria*

The exclusion criteria included: tumor patients, history of right limb fractures, deformities, epiphyseal plate unclosed, severe bone hyperplasia, and severe osteoporosis.

### *CT Data*

The CT data for the right distal supracondylar femurs were obtained from DICOM files using GE Medical Systems (Brightspeed, OPTIMA660). Scans (resolution, 512 × 512) were taken vertically to the long axis of the femur; we obtained 929–2140 continuous images <1 mm thick at the femoral epicondyle level and 10 cm above the knee joint.

The CT data were transferred to the computer from the mobile hard disk. Results for the cancellous bone and marrow cavity were retained, and information for the cortical bone was erased. Skeletal models were reconstructed with Mimics 17.0 software (Materialise, Haasrode, Belgium). The surfaces of the 3‐D reconstructed models were smoothed with Geomagic studio 12.0 software (Geomagic, Rock Hill, SC, USA), which was the second step in data processing. The last step was to imitate resection using the Solidworks 2014 software (Dassault Systemes S.A., Concord, MA, USA), which established a 3‐D coordinate system. The mechanical axis of the distal femoral shaft was defined as the Z‐axis; the resection plane was vertical to the Z‐axis.

### *Variables Examined*

**Figure 1:**
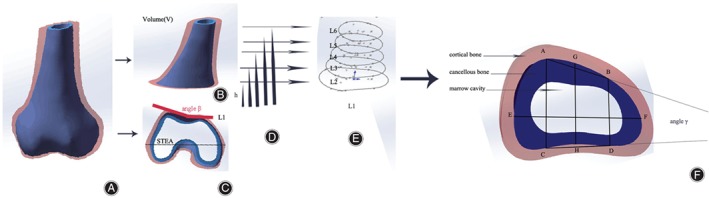
Schematic diagram of the measurement of the cortical lumen of the supracondylar femur. (A) The cortical lumen shape is reconstructed. (B) A model of the supracondylar femur is shown. Its volume is defined as volume V. (C) An osteotomy at the level of the femoral epicondyle, which was defined as the reference surface and which contacts the surgical epicondylar axis (STEA) and the angle of the patellar slide way (angle β). (D) The distance from each layer to the reference surface is defined as height h. (E) A model of the supracondylar femur divided into five layers. (F) EF is defined as the length of the transverse diameter at each osteotomy level. GH is the middle anteroposterior diameter. AC is the outside anteroposterior diameter. BD is the inside anteroposterior diameter. The angle between the front and back walls is defined as angle γ. Schematics are prepared using Photoshop software.

The variables examined were: the distance from layers to the reference surface, height (h), the length of the transverse diameter in each osteotomy level (EF), the middle anteroposterior diameter (GH), the medial and lateral anteroposterior diameters (AC and BD), the angle between the front and back walls (γ), and the reconstructed volume (V) of the cortical lumen. In addition, indicators were selected to classify the shape of the cortical lumen (Fig. 1).

### *Measurement Details*

The point O was defined as the posterior cruciate ligament in the proximal direction. The surgical epicondylar axis (STEA)^29^, ^30^, ^31^ was defined as the reference surface (L1), which was the most obvious at the region of the femoral epicondyle. The angle of the patellar trochlea was defined as β, and it was measured to position the range of the distal femur above the joint. When β = 180°, the bottom layer of the distal femur was named the second layer (L2). It is desirable to remove only the knee that contains the articular surface; the patella moving in the distal femur is in the trochanteric fovea, and the trochanteric fovea of the femur extends to the proximal of the supracondylar femur^32^, which is the part that needs to be retained as much as possible. Beginning with L_2_, the interval between adjacent layers was 10 mm, and L3, L4, L5, and L6 were named in turn. From L2 to L6, each layer of the X‐axis and the borderlines of the models intersected at E or F, located at the medial and lateral halves. The line connecting E and F was divided into four equal parts by three points. Through each point, a straight line was drawn perpendicular to EF. The vertical line nearest E intersected the front model's borderline at A and intersected the posterior model's borderline at C. The vertical line nearest F intersected the front model's borderline at B and intersected the posterior model's borderline at D. The vertical line located at the midpoint of the EF line intersected the front wall at G and the posterior wall at H. After these points were identified, we measured the length of AB, GH, AC, and EF in the different layers (i.e. L2–L6). The angle between BD and CD was defined as γ. The distance between the reference surface and the layer of interest was defined as height (h) and recorded for each layer. The volume of the 3‐D model of the cortical lumen containing L2–L6 was defined as V (Fig. 1), and V in each specimen‐specific 3‐D model was calculated using Geomagic Studio software in cubic centimeters.

### *Statistical Analysis*

The comparisons between the measurement date on the same level were assessed using Student's *t*‐test. Comparisons between the measurements of the different levels were made using the paired *t*‐test. The dimensions were summarized as mean ± standard deviation. A *P‐*value of <0.05 was considered significant. The bivariate correlations coefficient was represented as *r*. Linear regression analysis was used to explore the correlations between V and the measured dimensions. All statistical analyses were performed with SPSS 20.0 (IBM, Armonk, NY, USA).

## Results

### *Morphological Characteristics of Cortical Lumen of Supracondylar Femur*

The means and standard deviations (SD) for AC, BD, EF, GH, γ, and h in each layer and V are shown in Table 1.

**Table 1:** Characterization of the distal femoral specimens

Variable	L2	L3	L4	L5	L6
Valid, n (missing)	82 (0)	82 (0)	82 (0)	82 (0)	82 (0)
h, mm, mean (SD)	36.4 (9.05)	46.4 (9)	56.4 (9)	66.4 (9)	76.4 (9)
EF, mm, mean (SD)	40.88 (6.95)	34.76 (5.82)	30.64 (5.07)	27.65 (4.32)	25.53 (4.1)
GH, mm, mean (SD)	26.44 (3.16)	25.78 (3.57)	25.13 (3.03)	24.54 (3.06)	23.69 (2.86)
AC, mm, mean (SD)	29.65 (4.05)	27.39 (3.38)	26 (3.15)	24.68 (3.12)	23.47 (3.18)
BD, mm, mean (SD)	22.98 (3.45)	22.02 (2.97)	21.17 (2.94)	20.29 (3.18)	19.77 (2.83)
γ, °, mean (SD)	18.4 (4.76)	18.22 (4.91)	17.81 (4.8)	16.83 (5.8)	15.81 (6.49)

γ, the angle between the front and back walls; AC, the outside anteroposterior diameter; BD, the inside anteroposterior diameter; EF, the length of the transverse diameter; GH, the middle anteroposterior diameter; h, height of the supracondylar femur; L, layer (each layer is separated from the next by 10 mm); SD; standard deviation.

### *Correlations between Morphological Characteristics in Cortical Lumen*

#### *Comparisons of Indexes at Each Level*

At L2 (Table 2), there were strong correlations between V and EF2, GH2, AC2, and BD2 (*r* = 0.788, 0.818, 0.865, and 0.84, respectively; all *P* < 0.001). All length indexes (EF2, GH2, AC2, and BD2) were strongly correlated with each other (*r* range, 0.619–0.872). h2 was negatively correlated with V and EF2 (*r* = −0.248 [*P* < 0.05] and −0.631 [*P* < 0.001], respectively). γ2 was negatively correlated with BD2 and was not correlated with other indicators.

**Table 2:** Correlations between the various indicators in layer 2–6 and aspect ratios of each layer in the horizontal planes

Variable		L2	L3	L4	L5	L6
h*	V	−0.248 (0.025)	−0.248 (0.025)	−0.248 (0.025)	−0.248 (0.025)	−0.248 (0.025)
	EF	−0.631 (0.001)	−0.465 (0.001)	−0.351 (0.001)	−0.188 (0.091)	−0.163 (0.143)
	GH	−0.138 (0.215)	−0.041 (0.711)	0.125 (0.263)	0.107 (0.337)	0.047 (0.676)
	AC	−0.303 (0.006)	−0.101 (0.368)	−0.067 (0.549)	−0.089 (0.425)	−0.144 (0.197)
	BD	−0.158 (0.155)	0.049 (0.663)	0.11 (0.327)	0.093 (0.404)	0.098 (0.379)
	γ	−0.049 (0.759)	−0.123 (0.27)	−0.244 (0.027)	−0.385 (<0.001)	−0.506 (<0.001)
V*	EF	0.788 (<0.001)	0.847 (<0.001)	0.842 (<0.001)	0.785 (<0.001)	0.833 (<0.001)
	GH	0.818 (<0.001)	0.747 (<0.001)	0.844 (<0.001)	0.847 (<0.001)	0.779 (<0.001)
	AC	0.865 (<0.001)	0.894 (<0.001)	0.915 (<0.001)	0.917 (<0.001)	0.932 (<0.001)
	BD	0.84 (<0.001)	0.811 (<0.001)	0.799 (<0.001)	0.67 (<0.001)	0.758 (<0.001)
	γ	−0.192 (0.077)	−0.155 (0.16)	−0.035 (0.753)	0.1 (0.374)	0.275 (0.013)
EF*	GH	0.633 (<0.001)	0.535 (<0.001)	0.581 (<0.001)	0.567 (<0.001)	0.536 (<0.001)
	AC	0.794 (<0.001)	0.718 (<0.001)	0.695 (<0.001)	0.656 (<0.001)	0.712 (<0.001)
	BD	0.619 (<0.001)	0.539 (<0.001)	0.525 (<0.001)	0.398 (<0.001)	0.458 (<0.001)
	γ	−0.078 (0.466)	−0.065 (0.559)	0.004 (0.971)	0.150 (0.179)	0.340 (0.002)
GH*	AC	0.872 (<0.001)	0.794 (<0.001)	0.939 (<0.001)	0.934 (<0.001)	0.885 (<0.001)
	BD	0.82 (<0.001)	0.77 (<0.001)	0.952 (<0.001)	0.798 (<0.001)	0.884 (<0.001)
	γ	−0.083 (0.399)	−0.141 (0.205)	−0.151 (0.175)	−0.091 (0.415)	0.021 (0.851)
AC*	BD	0.78 (<0.001)	0.823 (<0.001)	0.88 (<0.001)	0.716 (<0.001)	0.855 (<0.001)
	γ	0.052 (0.711)	0.069 (0.539)	0.107 (0.338)	0.204 (0.066)	0.268 (0.015)
BD*	γ	−0.383 (<0.001)	−0.385 (<0.001)	−0.296 (0.007)	−0.258 (0.019)	−0.24 (0.030)
Mean (SD)						
AC/EF		0.7332 (0.0844)	0.7995 (0.1041)	0.8609 (0.1111)	0.9033 (0.1152)	0.9291 (0.1156)
GH/EF		0.6572 (0.0934)	0.7546 (0.1221)	0.834 (0.1238)	0.9002 (0.1279)	0.9424 (0.1361)
BD/EF		0.5701 (0.086)	0.6448 (0.1083)	0.7026 (0.1152)	0.7456 (0.1378)	0.7872 (0.1359)

γ, the angle between the front and back walls; AC, the outside anteroposterior diameter; BD, the inside anteroposterior diameter; EF, the length of the transverse diameter; GH, the middle anteroposterior diameter; h, height of the supracondylar femur; L, level; V, volume of the supracondylar femur. *mean r value (*P* value).

At L3 (Table 2), the length indexes (EF3, GH3, AC3, and BD3) correlated highly with V and each other, h3 correlated negatively with V and EF3, and γ3 correlated only with BD3 (*r* = −0.385, *P* < 0.05).

At L4 (Table 2), the correlations between V and length indexes or pairs of length indexes remained very high. There were negative correlations between γ4 and BD4 (*r* = −0.296, *P* < 0.001) and between γ4 and h4 (*r* = −0.244, *P* < 0.05).

At L5 (Table 2), h5 (*r* = −0.248, *P* < 0.05) and γ5 (*r* = −0.385, *P* < 0.001) correlated with V. Distinct from the results of the previous layers, there was no correlation between h and EF (*P* > 0.05). Correlations between V and lengths/pairs of length tended to weaken, although they remained strong (*r* = 0.785 [EF], 0.847 [GH], 0.917 [AC], and 0.67 [BD]). In addition, BD5 remained correlated with γ5 (*r* = −0.258, *P* < 0.05).

At L6 (Table 2), the correlations decreased between length indexes (*r* range, 0.458–0.885), and h6, V, EF6, AC6, and BD6 were significantly correlated with γ6. The absolute *r* value ranged from 0.24 to 0.34.

**Figure 2:**
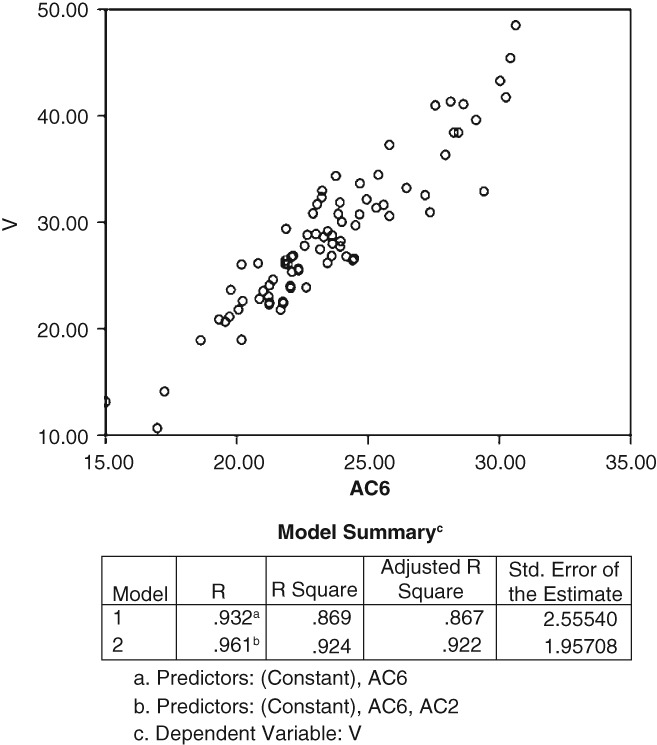
Correlation between the volume of the supracondylar femur (V) and the outside anteroposterior diameter of the sixth layer (AC6). The point distribution pattern indicates that the AC6 and V had a good linear correlation.

There were strong correlations between the length indexes of each layer and there was an obvious correlation between V and length index (Fig. 2).

#### *Correlations between Indexes and V*

The correlation between AC and V was always the largest correlation. The different indexes between each layer were collinear, and AC was the most sensitive indicator of V in each layer.

Linear regression analysis was used to explore the correlations between V and AC2, AC3, AC4, AC5, and AC6. Using the stepwise method, AC6 and AC2 were finally chosen as indicators, and two derived equations were obtained:

Model A: *R* ^2^ = 0.869 (*P* < 0.001). This model predicted 86.9% of the variation in V.

$$V\left({\mathrm{cm}}^{3}\right)=2.05\text{6AC}\_6\;\left(\mathrm{mm}\right)-19.629$$

Model B: *R* ^2^ = 0.922 (*P* < 0.001). This model more accurately explained 92.2% of the change in V.

$$V\left({\mathrm{cm}}^{3}\right)=0.63\text{2AC}\_2\;\left(\mathrm{mm}\right)+1.43\text{7AC}\_6\;\left(\mathrm{mm}\right)-23.865.$$

#### *Relevance of γ for anteroposterior diameter and height*

**Figure 3:**
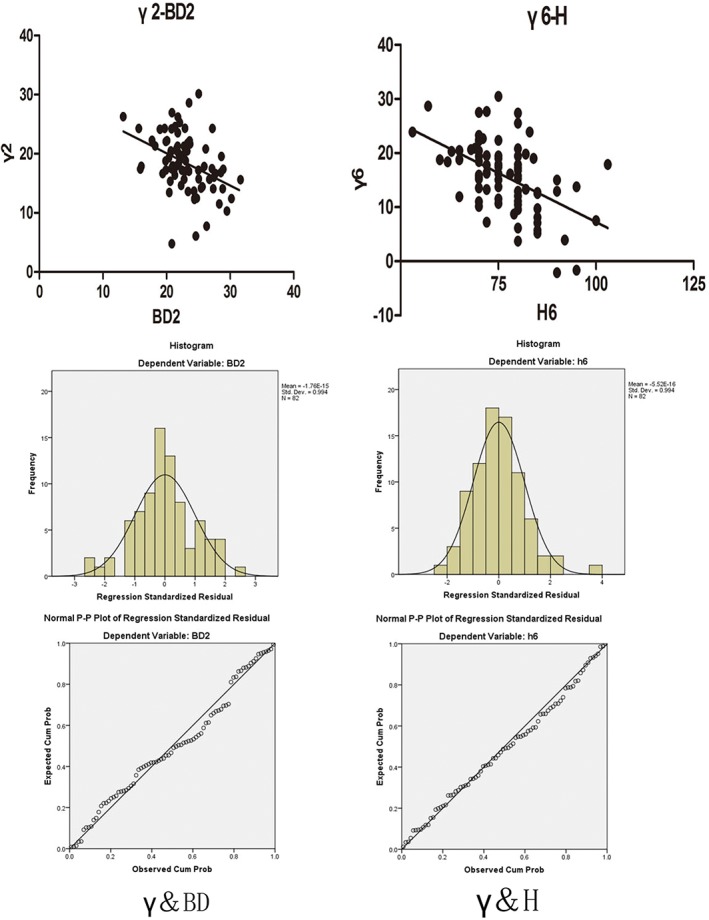
Correlation analysis: the angle between the front and back walls (γ) and the inside anteroposterior diameter (BD) of the second layer and the angle between the front and back walls and the supracondylar femoral height (H) of the sixth layer. The correlations between angle γ (that between the front and back walls) with the inside anteroposterior diameter (BD) and between angle γ with height h both followed normal distributions.

In layers 2, 3, and 4, γ correlated negatively with BD (*r* = −0.383, −0.385, and −0.296, respectively; all *P* < 0.001). In layers 5 and 6, γ correlated negatively with BD (*r* = −0.258 and 0.24, respectively; all *P* < 0.05). Thus, as BD increased, the absolute value of γ generally decreased (Fig. 3).

In accordance with layer order, h was not significantly correlated with γ in layers 1–3. In layer 4, a negative correlation appeared (*r* = −0.244, *P* < 0.05). In layers 5 and 6, the correlation was negative (*r* = −0.385, *P* < 0.001). Thus, angle γ was smaller as it became more proximal (Fig. 3).

### *Description of Shape of Horizontal Section*

We used aspect ratios as horizontal indicators for shapes in the horizontal plane. The aspect ratio uses the anteroposterior dimension (AC, GH, BD) of each layer divided by the inner and outer diameters (EF). From the results, we observed that the ratio of AC/EF and GH/EF increases from layer 2 to layer 6 and the value approaches 1 in the order from section 2 to section 6; AC/EF and GH/EF are both approximately 0.9 in section 5, suggesting that AC, GH, and EF are similar, with a disc‐like shape; BD/EF (0.57 <BD/EF <0.79), however, with the increase in the height of the cross‐section, shows a growing trend, but is not as large as that of AC/EF or GH/EF. In addition, BD is the shortest anteroposterior distance in each section, and the narrowest point is reflected in the shape (Table 2).

### *Description and Classification of Shape of Cortical Lumen*

**Figure 4:**
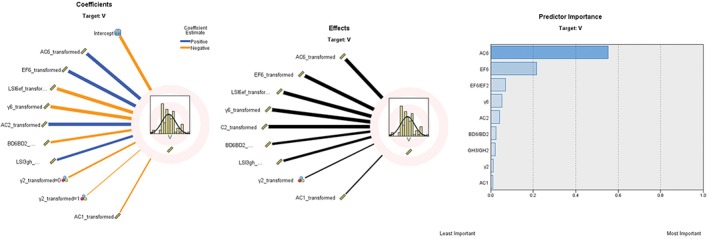
Coefficient effects and predictors of importance are shown in descending order with volume, V. The coronal width ratio was the most relevant ratio in the coronal plane. The sagittal width ratio was the most relevant ratio in the sagittal plane.

We sought to explain the morphological characteristics of the cortical lumen of the supracondylar femur and to select relevant predictors of volume. This approach was similar to that used by Noble *et al.*, who used length ratios of both sides to divide the proximal planar femoral medullary cavity into three types^12^. This method could be modeled two‐dimensionally because the 3‐D graphics could be converted into 2‐D coronal and sagittal planes. In each plane, a similar ratio could illustrate the projection's shape based on a normal distribution. Depending on the coefficient effect and the importance for prediction, EF6/EF2 and BD6/BD2 were selected as the lumen shrink indexes (LSI) at the coronal and sagittal planes and named LSIEF and LSIBD, respectively. The frequency description of the two LSI indexes for a normal distribution could be used to classify the distal femoral shape (Fig. 4).

**Figure 5:**
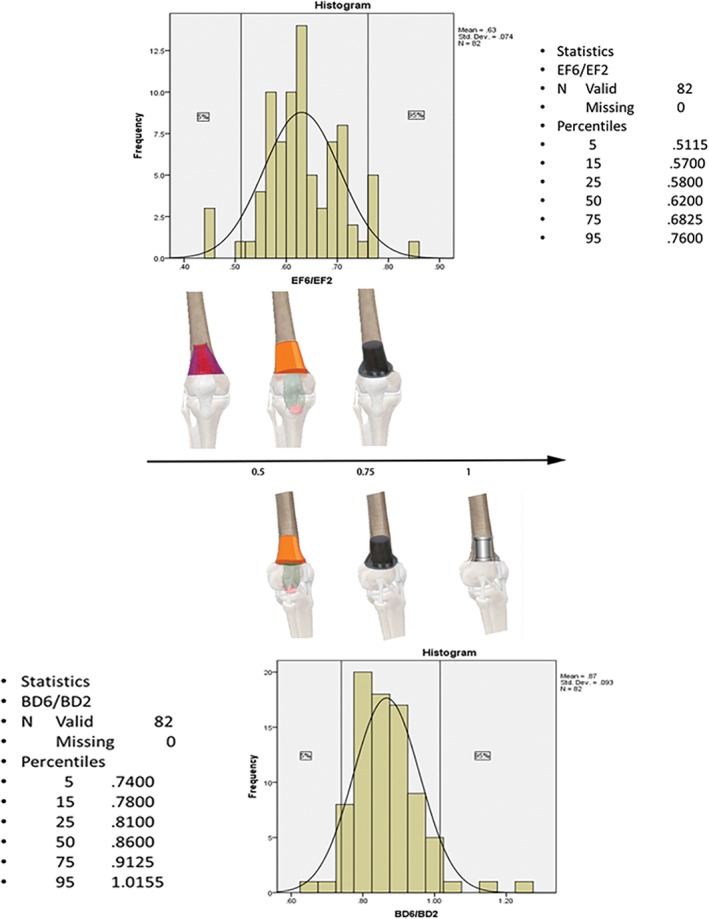
Classification of the cortical lumen of the supracondylar femur. The frequency distributions of the coronal width ratio in the coronal plane (LSIEF) (skirt, flowerpot, and top hat) and the sagittal width ratio in the sagittal plane (LSIBD) (flowerpot, top hat and stovepipe) divided into the three types.

According to the LSIEF distribution, the cortical lumen could be classified into 3 shapes: I‐type (skirt, <5%), J‐type (flowerpot, 5%–95%), and K‐type (top hat, >95%). LSI6BD (BD6/BD2) was adopted as the indicator for the coronal plane, and it could also be classified as J‐type (flowerpot, <5%), K‐type (top hat, 5%–95%), and L‐type (stovepipe, >95%) (Fig. 5).

**Figure 6:**
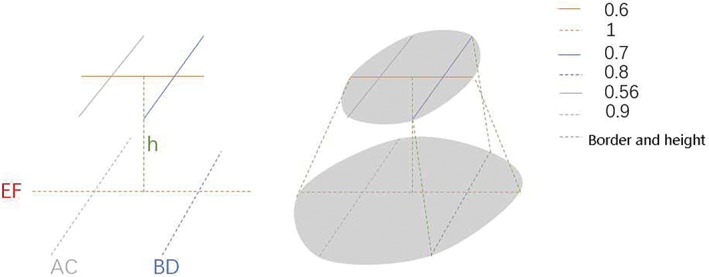
Drawing of 3‐D lumen of the supracondylar femur according to the ratio of each plane index ((i) h = 5 cm; (ii) EF2: AC2: BD2: EF6: AC6: BD6 = 1: 0.9: 0.8: 0.6: 0.56: 0.7).

Combining the distribution of sagittal (EF) and coronal (BD) LSI values, a combination of stereo (sagittal and coronal) and theoretical frequencies can be derived: IJ (0.25%), IK (4.5%), IL (0.25%), JJ (4.5%), JK (81%), JL (4.5%), KJ (0.25%), KK (4.5%), and KL (0.25%). According to the theoretical combination, the statistics of the actual combinations in this group are: 1.22%, 3.66%, 0%, 2.44%, 85.37%, 4.88%, 0%, 2.44%, 0%, which is in line with the theoretical frequency. According to the theoretical combination frequency, 81% of JK is the mainstream type, 18% of the sub‐mainstream types include JK, JJ, IK, and KK types, and rare types account for 1% of IJ, IL, KJ, and KL combinations. We selected the main shape combination of the coronal plane and sagittal plane and determined the ratio of several key factors according to each plane index to draw a 3‐D schematic diagram (Fig. 6). We used the following conditions: (i) h = 5 cm; (ii) EF2: AC2: BD2: EF6: AC6: BD6 = 1: 0.9: 0.8: 0.6: 0.56: 0.7; and (iii) smooth edge treatment.

## Discussion

In this study, morphological measurements of the cortical luminal of the femoral supracondylar were performed. The difference between the cortical luminal and the outer cortical shape is the absence or presence of cortex. Where cortical bone is thickened, the difference between the two is obvious. As far as we know, for morphological study, there is no explicit method to measure the cortical lumen. In previous research, the adjacent femoral condyles were measured by the length and ratio of the outer cortical shape^33^, ^34^, ^35^, ^36^. Therefore, we use a similar method to divide the section by height and record the length and ratio in each section of the femoral supracondylar. In the results, the volume (V)‐related parameters can be considered as a key point in the femoral supracondylar morphometric measurement. The following three results can be obtained. First, there were strong correlations between all of the length indexes in each layer and volume. Among all of length indexes, the inside anteroposterior diameter (AC) was the most sensitive indicator. Linear regression analysis was used to choose AC6 and AC2 as the key indicator of the volume. This indicator can be interpreted as the medial anteroposterior diameter in the horizontal cross‐section at both ends, which affected the shape of the femoral supracondylar.

Furthermore, the cross‐section shows a trend of a growing aseptic ratio with the increase of the height. The aseptic ratio BD/EF is not as large as AC/EF or GH/EF in the same cross‐section. Therefore, the lateral anterior–posterior diameter (BD) should be noted as the narrowest site. It is suggested that in the design of prosthesis the lateral semi‐anteroposterior diameter represented by BD should be strictly controlled to avoid fracture when an oversized prosthesis is pressed into the cortical luminal.

Finally, by comparison, LSIEF is the minimum value of LSI, which reflects that the coronal plane is the most obviously change with the increase of height. In the results for the sagittal plane, LSIEF and LSIBD are the most closely related to volume, so they were selected as key factors in the coronal and sagittal planes of the lumen morphology. These two indicators express the general shape of the cortical luminal in the plane in which they are located. The ratio, which reflects the shrinkage and flaring degree of the shape, was used to describe the scale size of the lengths of both ends.

The results indicated that the morphology of the cortical lumen of the femoral supracondylar is asymmetrical and irregular. However, there is still clinical significance for the morphological description. We take references from others. Just as CFI was used to divide the proximal medullary cavity into three types, the intramedullary nail was classified^37^. Based on the Notch shape index (NSI), the form of the intercondylar fossa has also been classified. NSI was adopted by Tillman *et al.* to compare intercondylar notch geometry between males and females. It was determined by dividing the width of the intercondylar notch by the height of the notch, which was classified into an inverted “U” and positive “A” shape^38^. This classification offered a visual description of the notch between the femoral condyles, which is significant in reconstruction of local bone and the cruciate ligament. We proposed that the shape of the supracondylar cortical luminal could be divided into three categories in the sagittal plane (flowerpot, top hat, and stovepipe) and the coronal plane (skirt, flowerpot and top hat). We analyzed the possibility of various combinations in the results, and found that the 3‐D shape type of the coronal flowerpot shape combined with the sagittal top hat shape is the most common in the cortical luminal of the femoral supracondylar, and this type can reach 81%. Other combinations occur at rates between 0.25% and 4.5%. Such division has a clinical significance in the design of the distal femoral prosthesis.

The morphology of the bone was important. The morphological matching degree of the prosthesis determines the stability in the implant–bone interface, which is also the main cause of prosthesis complications. Mckellop and Oconnor compared the biological effects of two kinds of femoral prostheses. Through the analysis of clinical effects, it was found that the aseptic loosening rate of the prosthesis with mismatched morphology was higher^39^, ^40^. To observe the initial stability of implant bone growth, Pilliar *et al.* compared the effect of implants in two groups of experiments on dogs and found that the gap between the prosthesis and bone after matching was less than 14 micra in order to achieve the bone growth^41^. Noble researched more ideal joint prostheses by means of biomechanics to reduce the incidence of complications in the whole hip joint, and concluded that reliable initial stability could be obtained only when the gap between the prosthesis and bone was less than 1 mm^42^. All the above studies have confirmed the importance of morphological matching, which can not only increase the stability of the prosthesis but also reduce complications, as well as increase the effect of bone ingrowth. If stability of the femoral supracondylar prosthesis can be achieved by morphological matching, this can also improve the rationality of mechanical distribution of the prosthesis to increase femoral supracondylar support.

One of limitations of our study was that we did not compare groups according to age and sex and distinguish between left and right legs. The second limitation was that the measurement was performed on the *in vivo* CT data, with differences in the physical measurements such as cadaver measurement. The third limitation was that a range of 5 cm of the femoral supracondylar was selected for measurement, which was a relative range.

### *Conclusions*

The supracondylar femur has an asymmetrical structural area. The most representative parameters were the medial femoral anteroposterior diameter (AC), the coronal width ratio (LSIEF), and the sagittal width ratio (LSIBD) with volume (V). Based on these parameters, the lumen shape of the supracondylar femur is gradually reduced as the height increases. The coronal plane is dominated by the flowerpot‐like morphology, and the sagittal plane is the narrowest in the lateral 1/3 and mainly resembles a top hat‐like morphology. Our results provide theoretical guidance for developing distal femoral prostheses and their clinical application.
